# The Killer Virus Called Nipah: A Review

**DOI:** 10.7759/cureus.3168

**Published:** 2018-08-21

**Authors:** Kamleshun Ramphul, Stephanie G Mejias, Vivian C Agumadu, Shaheen Sombans, Ruhi Sonaye, Petras Lohana

**Affiliations:** 1 Pediatrics, Shanghai Jiao Tong University School of Medicine/Shanghai Xin Hua Hospital, Shanghai, CHN; 2 Pediatrics, The University Iberoamericana Unibe School of Medicine/Robert Reid Cabral Children's Hospital, Santo Domingo, DOM; 3 Medicine, International University of the Health Sciences School of Medicine, Basseterre, KNA; 4 Internal Medicine, Bharati Vidyapeeth Deemed University Medical College and Hospital, Pune, IND; 5 Bharati Vidyapeeth Deemed University Medical College and Hospital, Thane, IND; 6 Medicine, Liaquat University of Medical and Health Sciences Hospital, Karachi, PAK

**Keywords:** nipah virus

## Abstract

Nipah virus (NiV) is a deadly virus with a high mortality rate that has affected many developing countries in the past. According to the Centers for Disease Control and Prevention (CDC), many economically deprived countries such as Madagascar, Cambodia, and Thailand are also at high risk for future outbreaks. The first case of NiV was reported in 1998 and almost two decades later, little scientific progress has been made in finding a proper treatment and prevention vaccine. As many developing countries are not properly equipped to fight the infection, it is vital to properly educate the health systems. The aim of this review is to provide an epidemiological background as well as to understand the transmission routes, presentation, and the diagnosis and prevention of this deadly virus.

## Introduction and background

The first case of Nipah virus (NiV) infection was initially reported in late September 1998 near Ipoh, West Malaysia. Over the months that followed, several clusters of infections were noted over different regions such as near Sikamet and Bukit Pelandok [1]. The cases were primarily assumed to be Japanese B Encephalitis and four patients were tested positive for the virus. However, the assessed victims were adults and not children, and there were reports of sick pigs with a barking cough, many of which died as well. These two features were not typical of Japanese B Encephalitis [2-3].

The virus was first isolated in March 1999 and based on its appearance, classified as a Paramyoviridae virus [4]. An outbreak occurred in 2001 in Meherpur, Bangladesh, and Siliguri, West Bengal, India and laboratory investigations that followed, failed to properly identify the organisms at that time. In 2014, the Philippines National Epidemiology Center was notified of deaths in Mindanao, Philippines. The patients were found positive for antibodies against NiV and immunoglobulin M (IgM) against NiV was also identified in three patients. A fatality of 53% was observed during that particular outbreak and 82% had acute encephalitis [5].

In May 2018, an outbreak of NiV was reported in the state of Kerala in India. It is believed that the human-to-human transmission during that epidemic was caused mostly by droplet infection. The 17th death occurred on May 30th and the State of Kerala announced that it has ended the monitoring on June 30th [6]. The National Centre for Disease Control India declared that a suspected case of Nipah infection is when “a person from a community affected by a NiV disease outbreak has fever with new onset of altered mental status or seizure and/or fever with headache and/or fever with cough or shortness of breath” and a confirmed case of Nipah infection is when a “suspected case has laboratory confirmation of NiV infection either by polymerase chain reaction (PCR) from respiratory secretions (throat swab), urine or cerebrospinal fluid or isolation of NiV from respiratory secretions (throat swab), urine or cerebrospinal fluid” [7].

According to the Centers for Disease Control and Prevention (CDC), up to now, many economically deprived countries with limited infrastructure and resources to prevent and combat the spread of this disease have been affected. Several countries such as Madagascar, Cambodia, and Thailand are also at risk for future outbursts of the disease. Proper understanding of the previous outbreaks and setting up of appropriate protocols can help protect these countries against future occurrences of NiV.

## Review

Method

We conducted a review based on published articles on NiV on MEDLINE and Google Scholar. The keywords included “Nipah virus,” “epidemiology of Nipah virus,” “transmission of Nipah virus,” “clinical manifestation of Nipah virus disease,” and “prevention of Nipah virus disease.” The search was limited to articles published from January 1st, 1998 to June 30th, 2018. All authors first reviewed the articles separately and then discussed them together.

What is Nipah virus?

The first analysis of the virus was carried out in early March 1999 by a team from the University of Malaysia. The cerebrospinal fluid of a patient suffering from encephalitis was analyzed and the virus was isolated and studied. The name Nipah virus was proposed as the source of the sample came from a region called Kampung Sungai Nipah [4]. From electron microscopy, the virus was found to have several characteristics similar to other Paramyxoviridae virus. The virally infected cells were observed to react strongly to Hendra virus antiserum. However, when antiserum from other Paramyxoviruses such as measles virus was used, it did not react at all. It was then hypothesized that Nipah and Hendra viruses might be closely related even if they are not identical. Upon examination, the NiV was seen to be pleomorphic and its size varied between 40 and 1900 nm. The shape can differ from spherical to filamentous and it can have a single layer of surface projections of an average 17 ± 1 nm. Two major lineages have been reported; NiV Malaysia and NiV Bangladesh. These two strains are very identical but they vary in length; the genome for NiV Malaysia is 18,246 nt in length and that of NiV Bangladesh is 18,252 nt [8].

How is Nipah virus transmitted?

Fruit bats in the genus Pteropus are the main reservoir of NiV [9-11]. Infected bats have not been reported to show any symptoms of the disease. More than 23 species of bats have been identified as possible reservoirs of NiV. Several species have served as host for the virus and these include naturally infected dogs, horses, pigs, cats, and humans [5, 12]. The mode of transmission between pigs involved direct contact via infected fluid such as urine or saliva. Multiple reports have also shown infections in guinea pigs, ferrets, and African green monkeys. Most Paramyxoviruses have a limited host range but NiV has shown a wider range of species tropism as it uses ephrinB2/B3 molecules to enter host cells [13].

Transmission to humans involves either direct bat-to-human transmission or from bats to human through an intermediate animal host. Human-to-human transmissions have also been reported in several outbreaks such as in Bangladesh and India [14-15].

**Figure 1:**
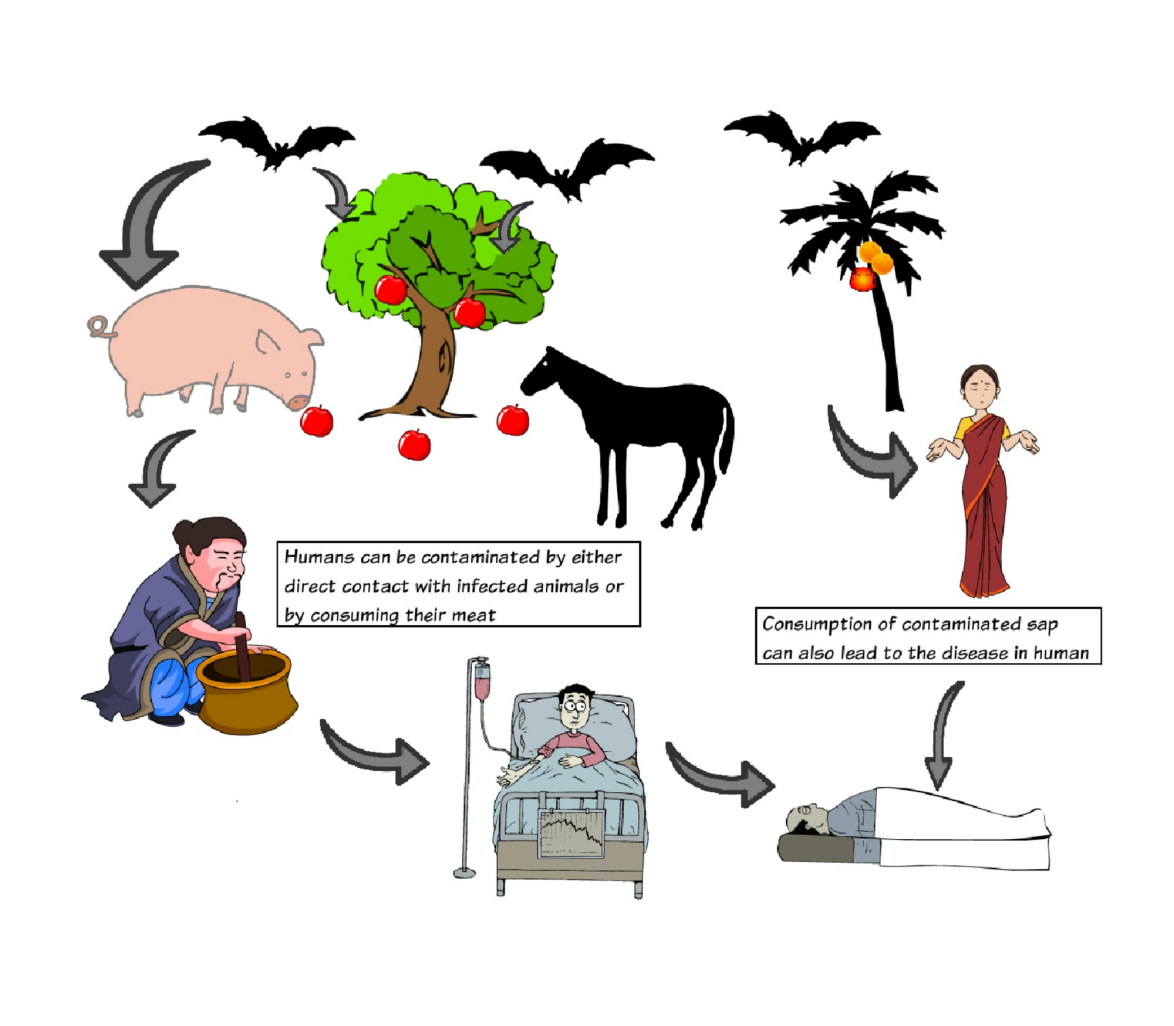
Pictorial description of different means of transmission of Nipah virus.

A summarized version is shown below in Figure 1.

What are the clinical findings of Nipah virus disease?

Nipah virus has an incubation period of seven to 40 days [1]. The disease starts with nonspecific symptoms such as sudden onset of fever, headache, and myalgia. Nausea and vomiting can also be present. One-third of the patients have shown meningismus. In 60% of cases, the patients rapidly deteriorated over the next five to seven days and had severe neurological symptoms. Some 20% were reported to have seizures from Nipah encephalitis. Other neurological symptoms such as tremors, areflexia, and segmental myoclonus were also observed. Long-term survivors have complained of persistent fatigue and daytime somnolence as well [16]. Tan et al. reported that in 160 cases who survived NiV encephalitis, 7.5% had relapses and 3.4% showed late-onset encephalitis [17].

How is Nipah virus disease diagnosed?

While the presence of antibodies against NiV is a reference standard for diagnosis, this technique is not used as Nipah has been classified as biosafety level four. An enzyme-linked immunoassay test has a high specificity for diagnosis. Another possible test includes polymerase chain reaction [18].

Autopsies performed on 32 victims of the Malaysian outbreak showed vasculitis resulting in disseminated microinfarction. Similar vasculitic lesions were seen in the heart, kidneys, and respiratory tract. Medium-sized blood vessels were more involved with fibrinoid necrosis and endothelial multinucleated syncytia [19].

Magnetic resonance imaging (MRI) scans of patients with NiV have shown multiple asymmetric focal lesions of less than 5 mm in the subcortical and deep white matter without surrounding edema [20-21].

How can Nipah virus disease be treated and what is the outcome?

There is currently no vaccine against NiV. The treatment options for patients with NiV include mostly supportive care. Chong et al. have reported a lower mortality rate with ribavirin therapy [22]. The mortality rate has varied greatly in different outbreaks; in Malaysia, it was 40% in 1999 while in Bangladesh and India it was close to 70%. In some regions of Bangladesh, a high mortality rate of more than 90% was also reported.

How to prevent future outbreaks of Nipah virus disease?

As the vaccine against NiV is still in the preclinical stage [23] and no other proper treatment options are available, the main aim of NiV management should focus on prevention. It is vital to properly educate the at-risk populations about the means of transmission of the virus.

Farm animals should not be allowed to eat fruits that have been exposed to bats. Raw palm sap is also a possible source of infection and consumption should be avoided to reduce the risk of bat-to-human transmission. However, this is a challenging step for some cultures. Proper precautions, protective clothing, and gloves ought to be used while handling any sick or dead animals or patients. With the current epidemic in Kerala, India, the National Centre of Disease Control of India has strongly advised strict and proper hand hygiene after coming into contact with any sick person or animal by using soap and water. They also advised against the consumption of raw palm sap or toddy. People were warned against eating half-eaten fruits, entering abandoned wells, and to properly handle dead bodies [7]. In January 2017, international governments and pharmaceutical companies formed the Coalition for Epidemic Preparedness Innovations (CEPI) to fund and promote research for a safe, affordable, and effective vaccine against the disease. It will, however, take years before a vaccine is approved for use on a large scale.

## Conclusions

Nipah virus is a deadly virus and has been reported in many underdeveloped countries with a high mortality rate for each outbreak. Two decades after the first reported case, there is still no improvement in treatment options or an effective vaccine. However, with proper education future outbreaks can be prevented and should be the number one priority in combating NiV outbreaks.
